# Legumain promotes tubular ferroptosis by facilitating chaperone-mediated autophagy of GPX4 in AKI

**DOI:** 10.1038/s41419-020-03362-4

**Published:** 2021-01-11

**Authors:** Chuan’ai Chen, Dekun Wang, Yangyang Yu, Tianyuan Zhao, Ningning Min, Yan Wu, Lichun Kang, Yong Zhao, Lingfang Du, Mianzhi Zhang, Junbo Gong, Zhujun Zhang, Yuying Zhang, Xue Mi, Shijing Yue, Xiaoyue Tan

**Affiliations:** 1 School of Medicine, State Key Laboratory of Medicinal Chemical Biology, Nankai University, Tianjin, 300071 China; 2 Dongfang Hospital of Beijing University of Chinese Medicine, Beijing, 100078 China; 3 Tianjin Key Laboratory of Modern Drug Delivery and High Efficiency, Tianjin University, Tianjin, 300072 China

**Keywords:** Necroptosis, Glomerulus, Acute kidney injury

## Abstract

Legumain is required for maintenance of normal kidney homeostasis. However, its role in acute kidney injury (AKI) is still unclear. Here, we induced AKI by bilateral ischemia-reperfusion injury (IRI) of renal arteries or folic acid in *lgmn*^WT^ and *lgmn*^KO^ mice. We assessed serum creatinine, blood urea nitrogen, histological indexes of tubular injury, and expression of KIM-1 and NGAL. Inflammatory infiltration was evaluated by immunohistological staining of CD3 and F4/80, and expression of TNF-α, CCL-2, IL-33, and IL-1α. Ferroptosis was evaluated by Acsl4, Cox-2, reactive oxygen species (ROS) indexes H_2_DCFDA and DHE, MDA and glutathione peroxidase 4 (GPX4). We induced ferroptosis by hypoxia or erastin in primary mouse renal tubular epithelial cells (mRTECs). Cellular survival, Acsl4, Cox-2, LDH release, ROS, and MDA levels were measured. We analyzed the degradation of GPX4 through inhibition of proteasomes or autophagy. Lysosomal GPX4 was assessed to determine GPX4 degradation pathway. Immunoprecipitation (IP) was used to determine the interactions between legumain, GPX4, HSC70, and HSP90. For tentative treatment, RR-11a was administrated intraperitoneally to a mouse model of IRI-induced AKI. Our results showed that legumain deficiency attenuated acute tubular injury, inflammation, and ferroptosis in either IRI or folic acid-induced AKI model. Ferroptosis induced by hypoxia or erastin was dampened in *lgmn*^KO^ mRTECs compared with *lgmn*^WT^ control. Deficiency of legumain prevented chaperone-mediated autophagy of GPX4. Results of IP suggested interactions between legumain, HSC70, HSP90, and GPX4. Administration of RR-11a ameliorated ferroptosis and renal injury in the AKI model. Together, our data indicate that legumain promotes chaperone-mediated autophagy of GPX4 therefore facilitates tubular ferroptosis in AKI.

## Introduction

Acute kidney injury (AKI) is a severe clinical syndrome manifested by rapid and possibly transient loss of renal functions^1^. Because of an aging population, increasing incidences of cardiovascular diseases, diabetes mellitus, and chronic kidney diseases, as well as expanding characterization of risk factors including sepsis, exposure to contrast media, and other nephrotoxins, the incidence of AKI is continuously increasing^2,3^. There is still no satisfactory treatment to attenuate AKI or accelerate recovery, resulting in mortality of AKI as high as 50%^4^. Elucidating the regulatory mechanisms underlying the loss and repair of tubular cells during AKI is required to develop effective therapeutic strategies and improve prognosis.

Tubular cell death is the major early event during the process of AKI, which is accompanied by inflammation due to chemokines and damage-associated molecular patterns from the dead and dying cells^5^. Distinct forms of cell death have been indicated to participate in the loss of tubular cells in AKI^2,6^. Ferroptosis, a newly recognized form of programmed cell death characterized by iron-dependent lipid hydroperoxide accumulation to lethal levels, has been reported to be of pathophysiological relevance in AKI^7–9^. In mouse models of AKI induced by either ischemia-reperfusion injury (IRI) or oxalate crystal, ferroptosis is directly involved in the synchronized necrosis of renal tubules^10^. Ferroptosis and its subsequent immunogenicity play the primary role in the kidney damage of folic acid-induced AKI^11^. Among the known regulatory factors involved in ferroptosis, GPX4 is the critical enzyme that reduces phospholipid hydroperoxides, acting as a gatekeeper for ferroptosis through selectively detoxifying lipid hydroperoxides^12^. Genetically inactive GPX4 leads to tubular cell death in a pathologically relevant form of ferroptosis and the occurrence of lipid-oxidation-induced AKI^13^. Modulating GPX4 abundance or activity results in the initiation and suppression of ferroptosis or sensitizing cells to ferroptosis^14,15^. A recent study revealed that activation of chaperone-mediated autophagy (CMA) was involved in the degradation of GPX4, and stabilizing GPX4 by inhibition of CMA reduced ferroptosis^16^. In this study, we found that asparaginyl endopeptidase legumain was involved in the degradation of GPX4 through CMA, affecting the process of ferroptosis during the progression of AKI.

Legumain is a conserved asparaginyl endopeptidase that is highly expressed in proximal tubular cells in the physiological state^17^. The birth and outward appearance of legumain knockout mice is normal. Renal histological abnormalities were first evident around 2 months of life, including hyperplasia of proximal tubular cells and abnormal mitotic figures. Interstitial fibrosis appeared by 3 months and glomerular cysts became evidence around 10 months. Glomerular filtration rate falls with increased urine albumin occurred in 6-month-old knockout mice, implying legumain is indispensable for the maintenance of normal kidney physiology and homeostasis^18^. Our previous data have demonstrated that M2 macrophage-derived legumain promotes degradation of the extracellular matrix, resulting in an anti-fibrotic effect in a mouse model of obstructive nephropathy. However, the context-dependent role of legumain during AKI is still unclear. In this study, through genetic engineering of murine models, we found that legumain deficiency ameliorated ferroptosis of tubular cells in AKI induced by either IRI or nephrotoxic folic acid. Furthermore, the beneficial role of legumain deficiency was mediated by the maintenance of cytosolic GPX4. We also found that legumain interacted with GPX4 and facilitated its autophagic degradation.

Taken together, our findings revealed that legumain is involved in the pathogenesis of AKI via regulating the degradation of major ferroptosis-protective factor GPX4, suggesting legumain as a therapeutic target for the treatment of AKI.

## Results

### Deletion of legumain appears renal protective effects in mice with renal IRI

**Fig. 1:**
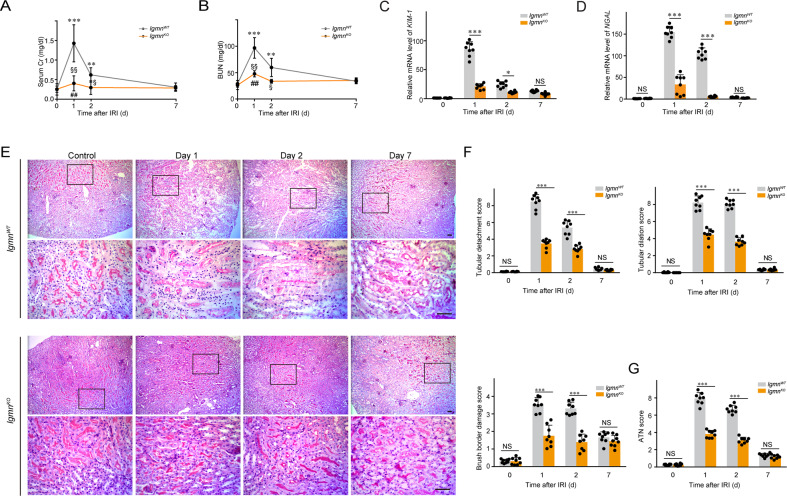
Legumain deficiency attenuates acute tubular injury in a mouse model of IRI. *Lgmn*^WT^ and *lgmn*^KO^ mice were randomized to 40 min of bilateral ischemia (*n* = 8). Kidney and serum samples were collected before and at day 1, 2, and 7 after IRI. **A**, **B** Renal functions assessed by serum creatinine (Cr) and BUN levels at the corresponding time points after IRI. ^*^P < 0.05, ^**^P < 0.01, ^***^P < 0.001 versus *lgmn*^WT^ control; ^♯^*P* < 0.05, ^♯♯^*P* < 0.01 versus *lgmn*^KO^ control; ^§^*P* < 0.05, ^§§^*P* < 0.01 versus *lgmn*^WT^ after IRI at the corresponding time point. **C**, **D** Quantification of kidney *KIM-1* and *NGAL* mRNA levels by qPCR. **E** Each pair of images includes a representative PAS-stained histologic photomicrograph of the kidney (top) from *lgmn*^WT^ and *lgmn*^KO^ mice on the indicated days after IRI and higher-magnification photomicrographs of the renal cortex (bottom; corresponding to the boxed area). Scale bar, 50 μm. **F** Quantification of histological renal damage. Tubular detachment, tubular dilation, and brush border damage were scored by the percentage area. **G** Histological ATN score. All data are expressed as the mean ± SD, ^*^P < 0.05, ^***^P < 0.001; two-way ANOVA.

Renal proximal tubular cells highly express legumain in the physiological state. To evaluate the effect of legumain in IRI-induced AKI, *lgmn* knockout mice (hereafter referred to as *lgmn*^KO^) and littermate control (*lgmn*^WT^) mice were subjected to 40 minutes of bilateral renal ischemia or sham surgery. BUN, serum creatinine, renal tubular injury, and transcript levels of *KIM-1* and *NGAL* were measured before and at 1, 2, and 7 days after reperfusion. A slight decrease of legumain was observed in *lgmn*^WT^ kidneys at day 1 after reperfusion, with an increase at day 2 and returned to the baseline level at day 7 (Supplementary Figure 1A). Compared with *lgmn*^WT^ control, IRI-induced increases in BUN and serum creatinine at 1 and 2 days after reperfusion were attenuated in *lgmn*^KO^ mice (Fig. 1A, B). Similarly, induced mRNA expression of molecular markers of tubular injury, *KIM-1* and *NGAL* was also suppressed in *lgmn*^KO^ mice (Fig. 1C, D). Based on the detachment and dilation of renal tubules and brush border damage shown by PAS staining, acute tubular necrosis (ATN) score was calculated. We found that indexes of tubular injury and ATN score at 1 and 2 days after reperfusion were decreased in the *lgmn*^KO^ group compared with *lgmn*^WT^ mice (Fig. 1E–G).

**Fig. 2:**
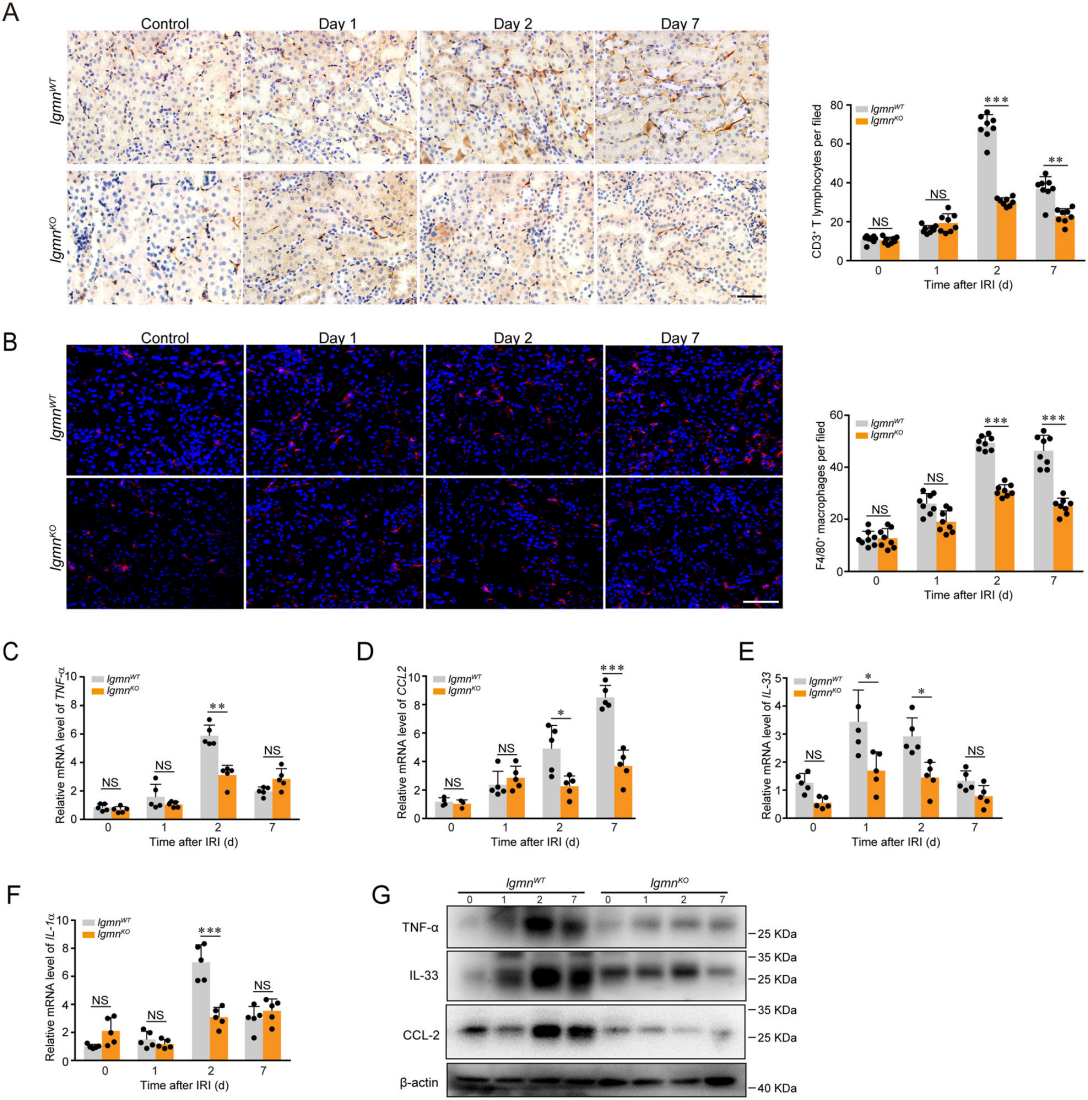
Legumain deficiency decreases inflammatory reactions in the mouse model of IRI. *Lgmn*^WT^ and *lgmn*^KO^ mice were randomized to 40 minutes of bilateral ischemia (*n* = 8). Collection of kidney samples was performed as described in Fig. 1. **A** Representative images (left panel) and quantification (right panel) of CD3-positive cells in AKI kidneys of *lgmn*^WT^ and *lgmn*^KO^ mice. Scale bar, 50 μm. **B** Representative confocal images (left panel) and quantification (right panel) of kidney sections stained for F4/80 (red) and with DAPI (blue). Scale bar, 50 μm. **C–F** qPCR analysis of mRNA expression of inflammatory genes *TNF-α*, *CCL-2*, *IL-33*, and *IL-1α* in kidneys of *lgmn*^WT^ and *lgmn*^KO^ mice. **G** Protein levels of inflammatory factor TNF-α, CCL-2, IL-33 in kidney samples assessed by western blotting. All data are expressed as the mean ± SD. ^*^P < 0.05, ^**^P < 0.01, ^***^P < 0.001; two-way ANOVA.

Inflammatory cell infiltration plays a key role in the pathogenesis of IRI-induced tubular lesions. Therefore, we compared the extent of inflammation in the IRI model of *lgmn*^WT^ and *lgmn*^KO^ mice. CD3-positive T cells and F4/80-positive macrophages were measured by immunohistological staining. Expression of proinflammatory cytokines *TNF-α*, *CCL2, IL-33*, and *IL-1α* was assessed. Our data showed that IRI-induced increase of inflammatory responses, including infiltration of inflammatory cells (Fig. 2A, B), and increase of inflammatory cytokines (Fig. 2C–G) were suppressed in *lgmn*^KO^ kidneys compared with control. Therefore, our data provide evidences that legumain promotes acute tubular lesions and inflammatory reactions induced by IRI.

### Legumain-deficient IRI mice exhibit reduced renal ferroptosis

**Fig. 3:**
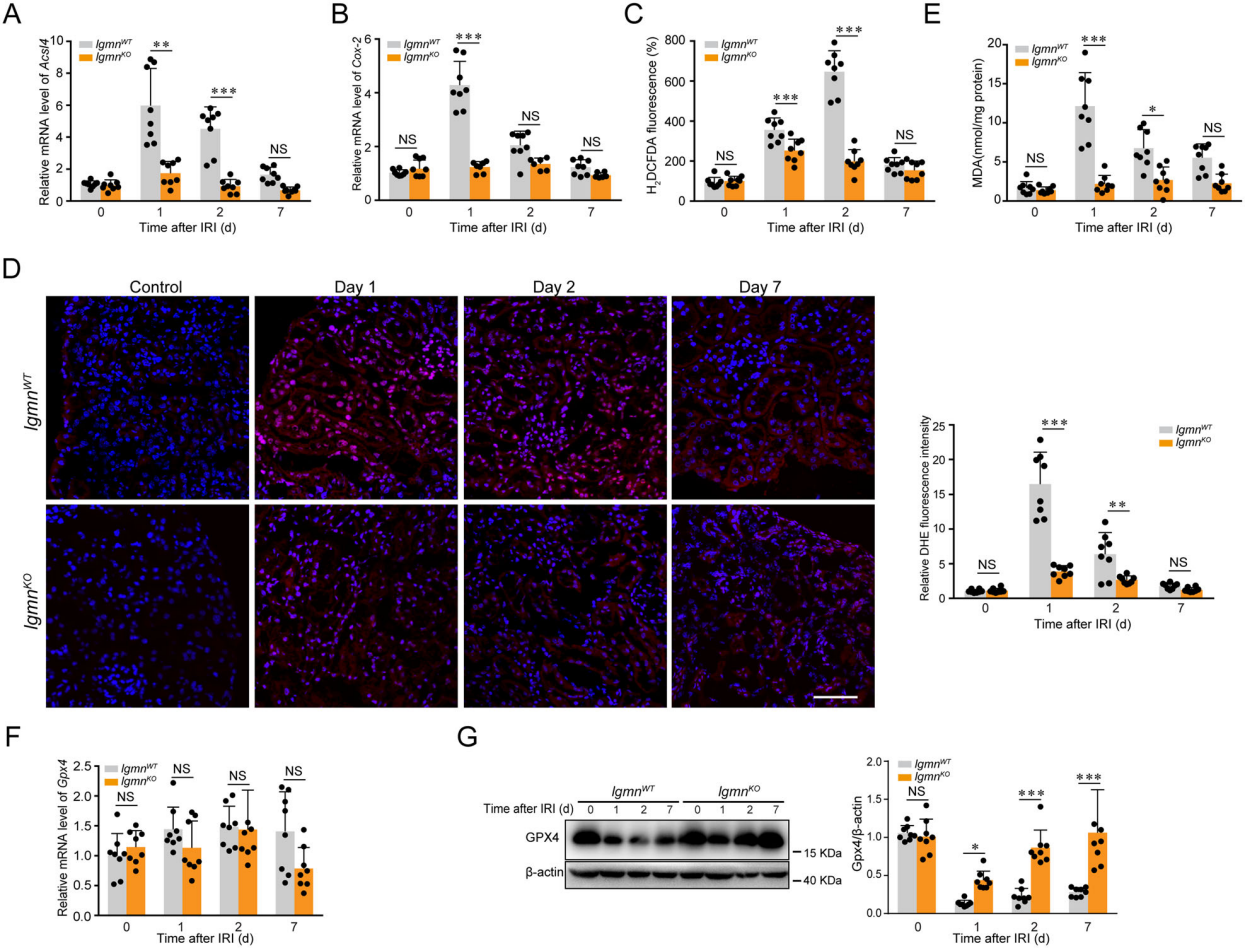
Legumain deficiency inhibits IRI-induced tubular ferroptosis. Collection of kidney samples was performed as described in Fig. 1 (n = 8). **A & B** Quantification of kidney *Acsl4* and *Cox-2* mRNA levels by qPCR. **C** Level of intracellular ROS in kidneys measured by the fluorescent probe carboxy-H_2_DCFDA. **D** Representative images of the fluorescence assay (left panel) and quantification (right panel) of ROS in kidneys using the fluorescent probe DHE (red), DAPI (blue) was used to stain the nuclei. Scale bar, 50 μm. **E** Levels of lipid peroxidation in kidneys were measured by MDA assay. **F** Quantification of kidney *GPX4* mRNA levels by qPCR. **G** Protein levels of GPX4 in kidney samples assessed by western blotting. Statistical results are shown in the right panel. All data are expressed as the mean ± SD. ^*^P < 0.05, ^**^P < 0.01, ^***^P < 0.001; two-way ANOVA.

Multiple insults result in tubular cell death in the context of IRI, including apoptosis, prominent programmed, and unprogrammed necrosis. It is recognized that ferroptosis, a unique form of programmed cell death characterized by iron-dependent and excess lipid peroxidation, contributes to renal tubular cell loss during AKI. To identify the role of ferroptosis in the reno-protective role of legumain knockout in the IRI model, we assessed indexes of ferroptosis, including products of lipid peroxidation, ROS, and degradation of phospholipid peroxidase GPX4. We found that Acsl4 and Cox-2, two enzymes responsible for the biosynthesis of ferroptosis-sensitive phospholipids, were inhibited in IRI *lgmn*^KO^ mice compared with control (Fig. 3A, B). Cytosolic ROS sensor carboxy-H_2_DCFDA and DHE were used to characterize intracellular ROS, and MDA content was measured to evaluate the end products of lipid peroxidation. Our results showed that ROS (Fig. 3C, D) and lipid peroxidation (Fig. 3E) were attenuated in *lgmn*^KO^ mice compared with control. Degradation of GPX4, a well-known inhibitory enzyme of lipid peroxidation is critical for driving ferroptosis. To determine whether legumain deficiency affected the transcription level of GPX4, we measured GPX4 mRNA in kidney samples and found that IRI did not influence mRNA level of GPX4 in *lgmn*^KO^ or *lgmn*^WT^ mice (Fig. 3F). However, IRI induced a reduction of GPX4 protein in *lgmn*^WT^ group, which was blocked in *lgmn*^KO^ mice (Fig. 3G). As for the markers of apoptosis and necroptosis, we found that there was no significant difference on cleaved caspase 3 at 1, 2, or 7-day of IRI and p-MLKL at 1 or 7-day of IRI between *lgmn*^WT^ and *lgmn*^KO^ mice, although less p-MLKL could be detected on day 2 of IRI in the *lgmn*^KO^ mice. (Supplementary Figure 2A, B). Collectively, these results indicate that legumain deficiency inhibits ferroptosis induced by IRI.

### Downregulation of legumain inhibits ferroptosis in renal tubular cells

**Fig. 4:**
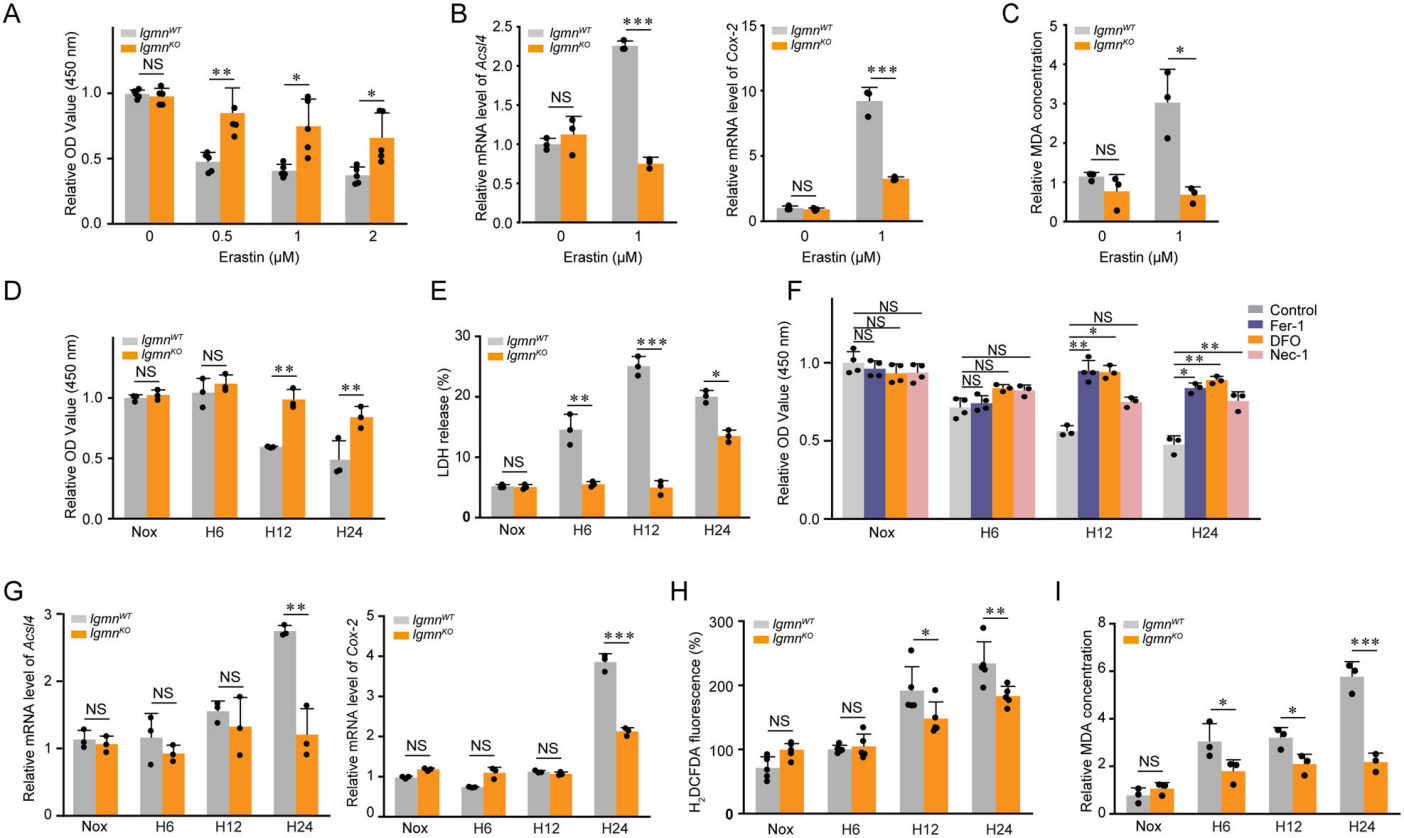
Downregulation of legumain suppresses ferroptosis in tubular cells. Primary mouse tubular epithelial cells were isolated from *lgmn*^WT^ and *lgmn*^KO^ mice. mRTECs were treated with 0.5, 1, and 2 μM erastin for 24 hours. **A** Cell survival was measured by CCK-8 assays. Data were collected from three independent experiments. **B** Quantification of *Acsl4* and *Cox-2* mRNA levels by qPCR. **C** Lipid peroxidation levels were measured by MDA assay. mRTECs were incubated under normoxic or hypoxic conditions for 6, 12, and 24 h. **D** Cell survival was measured by CCK-8 assays. **E** Cell lysates and supernatants were collected at the indicated time points for LDH release assays. **F** mRTECs were incubated under normoxic or hypoxic conditions for 6, 12, and 24 hours, with or without pretreatment of 2 hours of Fer-1 (1 μM), DFO (10 μM) or Nec1 (5 μM). Cell survival was determined 24 h thereafter using CCK-8 assays. **G** Quantification of *Acsl4* and *Cox-2* mRNA levels by qPCR. **H** Intracellular ROS levels were measured using the fluorescent probe carboxy-H_2_DCFDA. **I** Lipid peroxidation levels were measured by MDA assay. Data were collected from three independent experiments. All data are expressed as the mean ± SD. ^*^P < 0.05, ^**^P < 0.01, ^***^P < 0.001; two-way ANOVA.

To further identify whether legumain deficiency protects renal tubular cells against ferroptosis, we cultured primary mRTECs isolated from *lgmn*^WT^ and *lgmn*^KO^ mice. *lgmn*^WT^ and *lgmn*^KO^ mRTECs were treated with ferroptosis inducer erastin or simulated by hypoxia. Our results showed that both erastin and hypoxic stimuli induced legumain in mRTECs (Supplementary Figure 1B, C). Legumain knockout rescued erastin-induced decrease of cell survival and expression of *Acsl4* and *Cox-2* mRNAs (Fig. 4A, B). Increased MDA induced by erastin was also rescued after legumain knockout (Fig. 4C). Similarly, hypoxia reduced cell survival and increased LDH release, but these effects were blocked by knockout of legumain (Fig. 4D, E). Inhibitors of ferroptosis Fer-1 and iron chelator DFO rescued cell death induced by hypoxia of 12 and 24 h, while inhibitor of necroptosis Nec-1 only partially protect cell death from hypoxic injury of 24 h (Fig. 4F), indicating ferroptosis contributing to the hypoxia-induced cell death. We then detected ferroptosis indexes in *lgmn*^WT^ and *lgmn*^KO^ mRTECs stimulated by hypoxia. We found that hypoxia not only increased expression of *Acsl4* and *Cox-2* mRNAs, but also level of ROS and MDA. Compared with wildtype control, *Acsl4* and *Cox-2* mRNA expression (Fig. 4G) and level of ROS and MDA were reduced in *lgmn*^KO^ mRTECs (Fig. 4H, I). Similarly, legumain knockout mitigated ferroptosis induce by erastin or RSL3 in 786-O and OSRC-2, two renal tubular carcinoma cell lines (Supplementary Figure 4). Taken together, our data indicate that downregulation of legumain inhibits erastin- and hypoxia-induced ferroptosis in renal tubular cells.

### Legumain participates in lysosomal degradation of GPX4 during ferroptosis

**Fig. 5:**
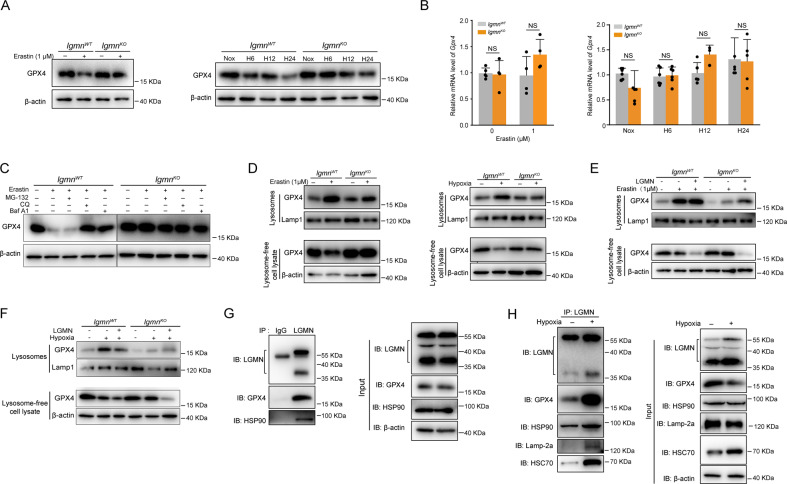
Legumain promotes chaperone-mediated autophagy of GPX4. Primary *lgmn*^WT^ and *lgmn*^KO^ mRTECs were treated with 1 μM erastin for 24 hours or incubated under normoxic or hypoxic conditions for 6, 12, and 24 hours. **A** Western blot assay of GPX4. **B** Quantification of *GPX4* mRNA levels by qPCR. Primary *lgmn*^WT^ and *lgmn*^KO^ mRTECs were pretreated with MG132, CQ, and Baf A1 for 2 hours before treatment with 1 μM erastin for 24 h. **C** Western blot using an antibody against GPX4. **D** mRTECs were treated with 1 μM erastin for 24 h or incubated under hypoxic conditions for 24 h. Lysosomes and lysosome-free cell lysates were collected and the protein level of GPX4 was measured by western blotting. Lamp1 and β-actin were used as loading controls of lysosomal and non-lysosomal proteins, respectively. **E**, **F** Primary mRTECs from *lgmn*^WT^ and *lgmn*^KO^ mice were infected with control or legumain-overexpressing lentiviral constructs for 48 h and then treated with 1 μM erastin or incubated under hypoxic conditions. Western blotting was performed using an antibody against GPX4. **G** In cultured HK-2 cells, a co-IP assay was performed using an antibody against legumain or control IgG and western blotting for legumain, GPX4, Lamp-2a, HSC70, and HSP90 was performed. **H** HK-2 cells were incubated under normoxic or hypoxic conditions for 24 h. Co-IP assays were performed using an antibody against legumain or control IgG and then western blotting for legumain, GPX4, Lamp-2a, HSC70, and HSP90 was performed.

Given that legumain plays a role in tubular ferroptosis, we further explored the underlying molecular mechanism. Both erastin and hypoxia inhibited GPX4 protein in wildtype primary tubular cells, and this effect was rescued in legumain knockout cells (Fig. 5A). No significant difference of GPX4 mRNA was found between legumain knockout and wildtype cells (Fig. 5B), suggesting that the regulatory effect of legumain on GPX4 occurs at protein level. Various protein degradation inhibitors, including proteasome and lysosome inhibitors, were used to evaluate the role of distinct degradation pathways in the legumain-related change of GPX4 level. Treatment with lysosome inhibitors chloroquine or bafilomycin A1, but not proteasome inhibitor MG-132, rescued the decrease of erastin-induced GPX4 (Fig. 5C). Similar results were found in erastin or RSL3 induced ferroptosis in 786-O and OSRC-2 cells **(**Supplementary Figure 5). These results suggest that the protective effect of legumain deficiency on GPX4 dependents on inhibition of its lysosomal degradation.

We further compared GPX4 in lysosomes and other cellular compartments during erastin- and hypoxia-induced ferroptosis in *lgmn*^KO^ and *lgmn*^WT^ mRTECs. Our results showed that knockout of legumain abolished increase of lysosomal GPX4 induced by erastin or hypoxia (Fig. 5D). Moreover, overexpression of legumain blocked the change of GPX4 induced by legumain knockout (Fig. 5E, F). A previous study has indicated that activation of ferroptosis leads to increases of HSC70-mediated lysosomal delivery and degradation of GPX4. Therefore, we hypothesized that cytosolic legumain participated in the chaperone-mediated lysosomal transport of GPX4. Immunoprecipitation assays suggested interactions between legumain, HSP90, HSC70, lamp-2a and GPX4 (Fig. 5G). These interactions were increased by hypoxia compared with control (Fig. 5H). Therefore, our data provide evidences that cytosolic legumain interacts with HSC70, HSP90, lamp-2a and GPX4, mediating lysosomal transport of GPX4 and subsequently ferroptosis.

### Legumain deficiency attenuates tubular ferroptosis in folic acid-induced AKI

**Fig. 6:**
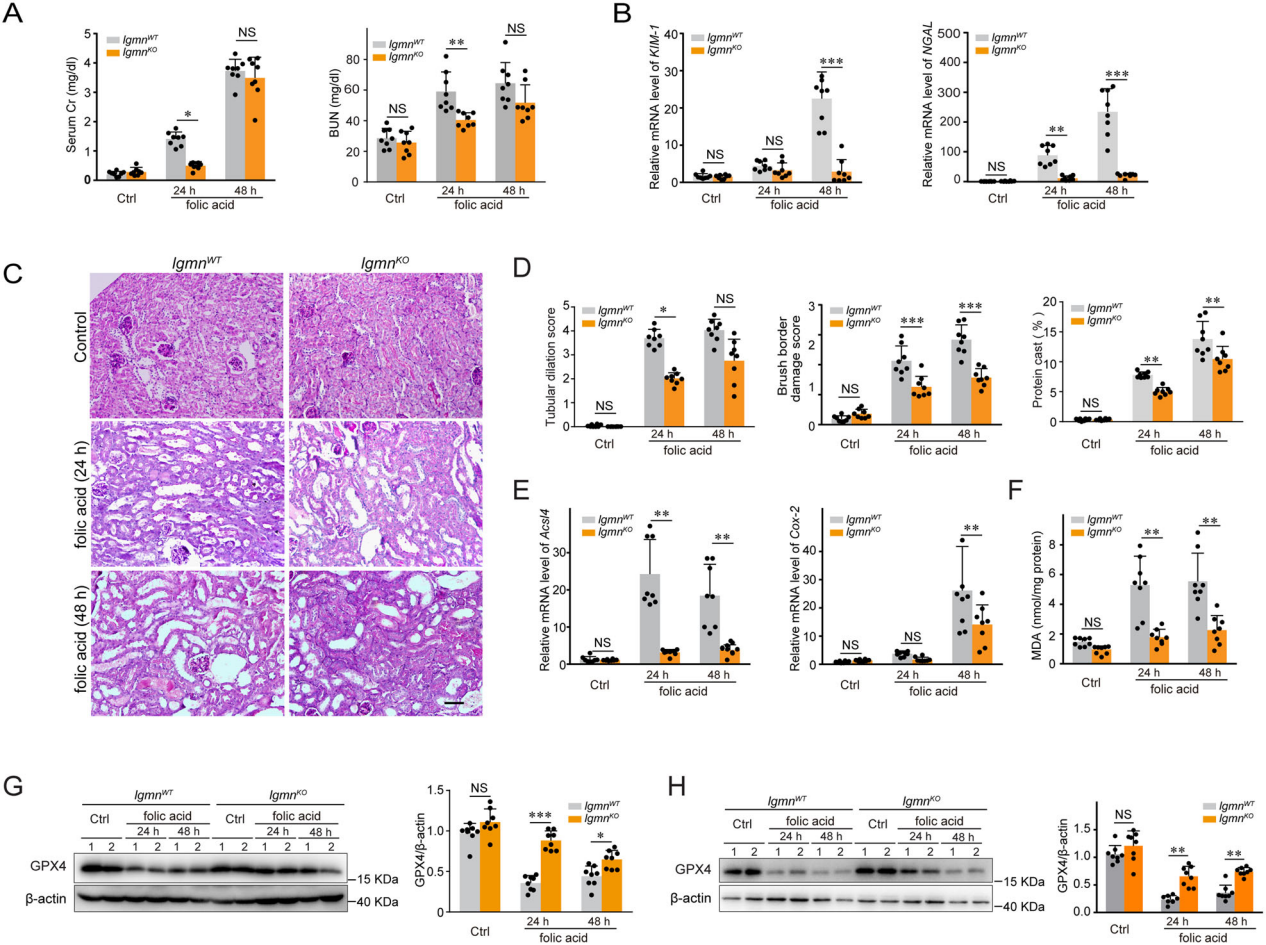
Legumain deficiency attenuates tubular injury and ferroptosis in folic acid-induced AKI. Folic acid was administrated intraperitoneally to *lgmn*^WT^ and *lgmn*^KO^ mice, and samples were collected at 24 and 48 hours after injection (n = 8) **A** Renal functions assessed by serum creatinine and BUN levels. **B** Quantification of kidney *KIM-1* and *NGAL* mRNA levels by qPCR. **C** Representative images of PAS-stained kidney sections. Scale bar, 50 μm. **D** Quantification of histological renal damage. Tubular dilation and brush border damage are scored, and protein casts are presented as percentage areas. **E** Quantification of kidney *Acsl4* and *Cox-2* mRNA levels by qPCR. **F** Lipid peroxidation levels in kidneys were measured by MDA assay. **G** Protein levels of GPX4 in kidney samples assessed by western blotting. Right panel shows the statistical data. **H** Primary renal tubular epithelial cells were isolated from *lgmn*^WT^ and *lgmn*^KO^ mice after injection of folic acid and the protein level of GPX4 was quantified by western blotting. Left panels show representative images and the right panel shows the statistical data. All data are expressed as the mean ± SD. ^*^P < 0.05, ^**^P < 0.01, ^***^P < 0.001; two-way ANOVA.

We next investigated the effect of legumain deficiency on nephrotoxic folic acid model in which ferroptosis has been proven to be critical for the pathogenesis of AKI. We found that legumain was increased in 24 and 48 h of folic acid-induced AKI model (Supplementary Figure 6A, B). In vitro, treatment of folic acid increased level of legumain in cultured mRTECs (Supplementary Figure 6C). Compared with wildtype control, lower levels of serum creatinine and BUN were found in *lgmn*^KO^ mice (Fig. 6A). Consistently, folic acid-induced increases of *KIM-1* and *NGAL* expression were also suppressed in *lgmn*^KO^ mice (Fig. 6B). Dilation of renal tubules, brush border damage, and the protein cast percentage assessed by PAS staining showed similar results, indicating that knockout of legumain attenuated folic acid-induced tubular injury (Fig. 6C, D). Indexes of ferroptosis, including expression of *Acsl4* and *Cox-2* mRNAs and level of MDA, were significantly increased after 24 and 48 hours of treatment of folic acid. Compared with control, both *Acsl4* and *Cox-2* mRNAs and level of MDA were reduced in knockout mice (Fig. 6E, F). The decreased GPX4 level in kidney tissue after 24 and 48-hour of treatment with folic acid was reserved in knockout mice compared with control (Fig. 6G). In mRTECs isolated from the folic acid-induced AKI model, similar changes in GPX4 expression were found. Specifically, the change in the GPX4 level of *lgmn*^KO^ mRTECs after 24 and 48-hour of folic acid treatment was reserved compared with the control (Fig. 6H). Thus, our data demonstrate that knockout of legumain also protects against tubular lesions and ferroptosis caused by nephrotoxic folic acid.

### RR-11a ameliorates ferroptosis and acute tubular injury in the IRI model

**Fig. 7:**
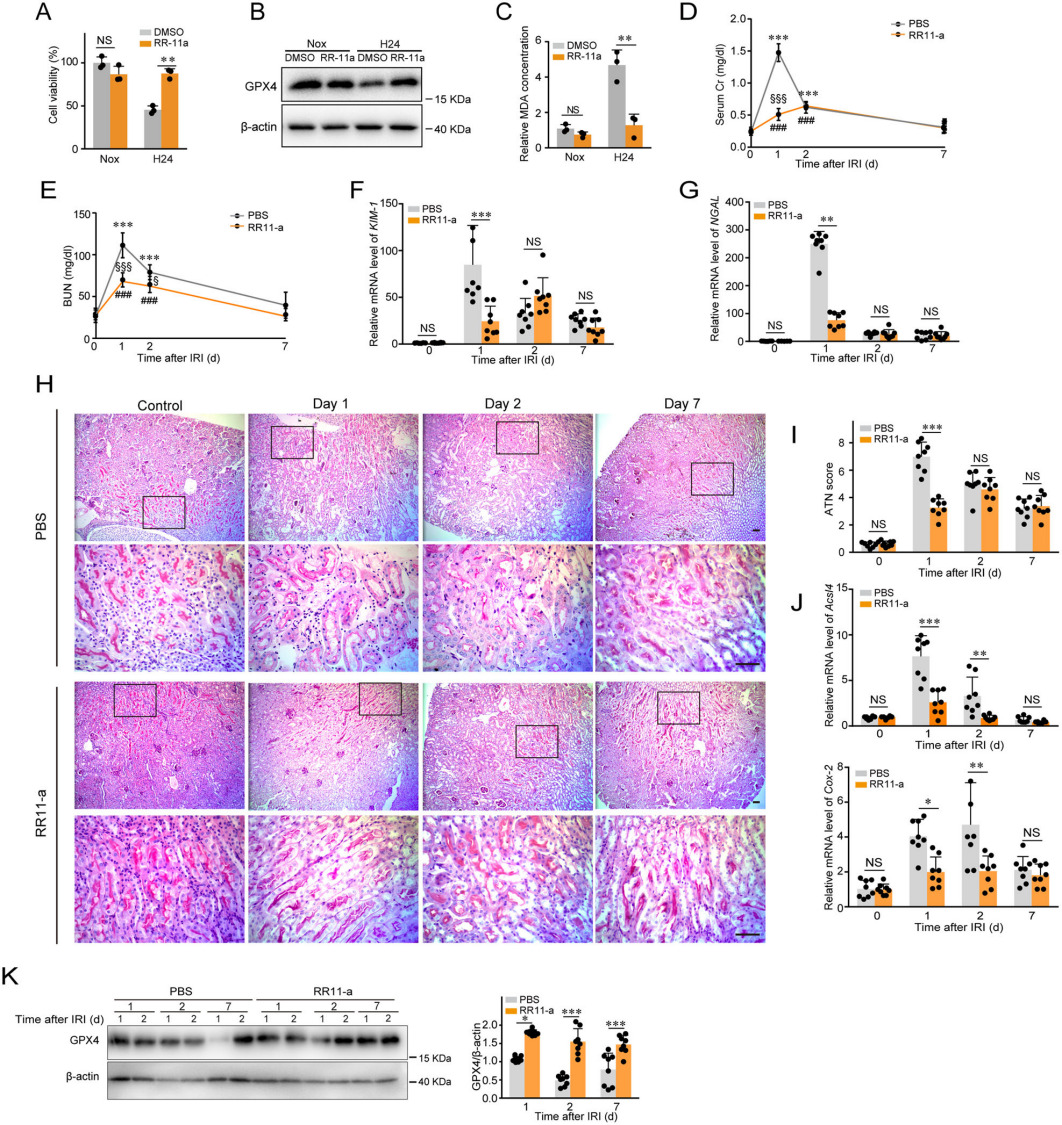
Legumain inhibitor RR-11a attenuates ferroptosis and tubular injury induced by IRI. Primary *lgmn*^WT^ mRTECs were pretreated with RR-11a (100 nM) for 2 h before incubating under hypoxic conditions for 24 h. **A** Cell survival was measured by CCK-8 assays. **B** Western blot assay of GPX4. **C** Lipid peroxidation levels were measured by MDA assay. *Lgmn*^WT^ mice were i.p. injected with RR-11a or the vehicle control at 40 min before bilateral ischemia (*n* = 8). Kidneys and serum were collected before and at day 1, 2, and 7 after IRI. **D**, **E** Renal functions assessed by serum creatinine and BUN levels. ^**^P < 0.01, ^***^P < 0.001 versus vehicle control group before IRI; ^♯♯^*P* < 0.01, ^♯♯♯^*P* < 0.001 versus RR-11a group before IRI; ^§^*P* < 0.05, ^§§^*P* < 0.01, ^§§§^*P* < 0.001 versus vehicle control group after IRI at the corresponding time point. **F**, **G** Quantification of kidney *KIM-1* and *NGAL* mRNA levels by qPCR. **H** Each pair of images includes a representative PAS-stained histologic photomicrograph of the kidney (top) and higher-magnification photomicrographs of the renal cortex (bottom; corresponding to the boxed area). Scale bar, 50 μm. **I** Quantification of acute tubular necrosis. **J** Quantification of kidney *Acsl4* and *Cox-2* mRNA levels by qPCR. **K** Western blot assay using the antibody against GPX4. Right panel shows the statistical data. Data were collected from three independent experiments. All data are expressed as the mean ± SD. ^**^P < 0.01; two-way ANOVA. All data are expressed as the mean ± SD. ^*^P < 0.05^, **^P < 0.01, ^***^P < 0.001; two-way ANOVA.

To investigate the potential application of legumain inhibitor in AKI, we firstly confirmed the inhibitory effect of RR-11a on ferroptosis of mRTECs induced by hypoxia in vitro. (Fig. 7A–C). Then we determined whether administration of RR-11a ameliorated acute tubular injury in the IRI model. At 1 day after IRI, levels of serum creatinine and BUN were significantly lower in the RR-11a group compared with the vehicle control (Fig. 7D, E). Indexes of tubular injury, including mRNA expression of *KIM-1* and *NGAL* and the histological ATN score were improved in the RR-11a group (Fig. 7F–I). To examine changes in ferroptosis, we compared mRNA expression of *Acsl4*, *Cox-2*, and GPX4 at various time points after IRI. The results showed that administration of RR-11a abolished the increases of *Acsl4* and *Cox-2* expression and suppression of GPX4 at 1, 2, and 7 days after IRI **(**Fig. 7J, K). Therefore, our data show that the inhibitor of legumain attenuates tubular injury and ferroptosis induced by IRI.

## Discussion

Progress on developing therapeutic strategies that improve AKI outcomes remains slow, although a number of candidate molecular targets for effective therapies to prevent or ameliorate AKI have been identified in recent years. Our study showed that depletion of legumain, a conserved lysosomal endopeptidase, protects against the tubular injury in AKI caused by IRI or nephrotoxic folic acid. Moreover, we provide evidences that the beneficial effect of legumain deficiency correlates with suppression of tubular ferroptosis.

As a unique pattern of programmed cell death different from apoptosis, ferroptosis has been shown to be a promising therapeutic target for kidney diseases, especially those characterized by tubular death, including AKI^19,20^. Novel small molecules that specifically block ferroptosis, including ferrostatin-1 and liproxstatin-1, protect isolated renal tubules from erastin- and iron loading-induced cell death, and reduce kidney injury following acute oxalate-induced damage and in a model of severe kidney IRI^21^. Compared with other forms of regulated cell death, ferroptosis results from inactivation of an essential metabolic process, leading to iron-catalyzed, lipid reactive oxygen species-mediated cellular collapse^14^. Cells undergoing ferroptosis are likely to release several immune modulators that are beneficial to trigger immune responses^5,7^. In this study, compared with wildtype control, IRI induced less infiltrated inflammatory cells and immunostimulatory signals in legumain knockout mice, which were accompanied by inhibition of ferroptosis. Thus, these data suggest that the renal beneficial effect of legumain knockout was not only due to the decrease of tubular cell loss, but also suppressed inflammation that amplifies injury responses and repair. It is noteworthy that studies have implicated distinct forms of regulated necrosis in AKI, indicating the necessity of combined therapies that block multiple pathways of regulated cell death simultaneously^2,10^.

Legumain is a member of the clan CD family C13 of cysteine proteases because of recognition of a conserved His^148^-Gly-spacer-Ala-Cys^189^ motif^22^. It has been shown to be involved in various pathological situations including cancer^23^, neurodegenerative disorders^24^, and kidney diseases^18^. The canonical translocation pathway of legumain is routed from the endoplasmic reticulum and Golgi to the endosome before it becomes activated in lysosomes^17^. However, legumain is also found at other “non-canonical” compartments outside of acidic vesicles, such as the cytoplasm, nucleus, and cell surface, implying diversity of its functions^25^. Loss of legumain induces proliferative abnormalities in the murine kidney due to accumulation of low molecular weight proteins in lysosomes, especially EGF receptor^18^. However, tubular cell- and macrophage-derived legumain has been reported to participate in remodeling of the extracellular matrix in the obstructive nephropathy model of unilateral ureteral obstruction^26,27^. In neurodegenerative disorders, the effects of legumain are mediated by cleavage of Tau and α-synuclein in Alzheimer’s and Parkinson’s diseases^24,28,29^. Studies in tumor condition indicate that cytosolic legumain interacts with E3 ligase TRAF6 and activates lys63-linked ubiquitination. This ubiquitination results in ternary complex formation with heat shock protein 90 (HSP90), thereby increasing intracellular legumain stability and secretion^30^. The concentration of legumain in circulation correlates positively with the malignancy of breast cancer patients^30^. The exact roles of cytosolic legumain in distinct contexts are still unclear since the pH value of the cytosol is obviously unsuitable for the maturation of enzymatic legumain. In the present study, we found that cytosolic legumain interacted with the ferroptosis inhibitor GPX4, thereby promoting ferroptosis by increasing lysosomal autophagy of GPX4.

As an enzyme crucial to convert toxic lipid hydroperoxides into nontoxic lipid alcohols, GPX4 has been thought to be the main controller of ferroptosis^12,31^. Inactivation of GPX4 by direct inhibition with RSL3 or depletion of GPX4 results in overwhelming lipid peroxidation and cell death^32,33^. A recent study showed that chaperone-mediated autophagy is involved in the execution of ferroptosis, and HSP90 acts as a common regulatory mechanism shared by necroptosis and ferroptosis^16^. Here, our data indicated interactions of legumain, GPX4, and HSP90 in the cytoplasm. In addition, knockout of legumain impeded lysosomal transport and subsequent autophagy of GPX4 in erastin- and hypoxia-induced ferroptosis of tubular cells. In different AKI models, autophagy is rapidly induced in proximal tubular cells^34^. Moreover, it has been implicated in tubular atrophy observed in post-AKI tubules^35,36^. Pharmacological or genomic blockade of autophagy induction in AKI enhances kidney injury^37–39^. At present, the effect of autophagy on kidney injury during AKI is still controversial. It is possible that the timing of autophagy is important to determine its beneficial or deleterious effects. Further study is obviously needed to elucidate the role of this process of degradation and recycling of intracellular substances in tubular injury caused by AKI.

In conclusion, our findings reveal that legumain is involved in the pathogenesis of AKI by participating in chaperone-mediated autophagy of critical ferroptosis-protective factor GPX4, suggesting legumain a therapeutic target and early diagnostic marker for AKI.

## Materials and methods

### Chemical reagents

Erastin, ferrostatin-1 (Fer-1), and necrostatin-1 (Nec-1) were purchased from MedChem Express (Monmouth Junction, NJ). Chloroquine (CQ) and bafilomycin A1 (Baf A1) were purchased from Selleck Chemicals (Houston, TX). MG-132, deferoxamine (DFO), and folic acid were obtained from Sigma-Aldrich (St. Louis, MO). RR-11a was purchased from Wuxi Apptech (Beijing, China).

### Animal models

The experimental animals were provided by Cyagen Biosciences, Inc. (Suzhou, China). The *lgmn*^KO^ mice were generated using homologous recombination methods. The genetic background of embryonic stem cells and the Flp mice used in this experiment was C57BL/6. Offspring were genotyped by PCR according to the manufacturer’s protocol (Cyagen). Littermates wild type mice were used as control mice (*lgmn*^WT^). Experiments were performed in accordance with the Institutional Animal Use and Care Committee of Nankai University. Two AKI models were established as follows. Mice were randomly separated into experimental groups and control groups. (1) Bilateral IRI: mice (male, 8–10 weeks old) on the *lgmn*^KO^ background or littermate control mice were anesthetized by an intraperitoneal (i.p.) injection of chloral hydrate and placed on a warm pad to retain their body temperature. A bilateral flank incision was made, both sides of the renal vessels were occluded with clamps for 40 min followed by removing the clamps to induce blood reperfusion. The same procedure was performed in the control group without vessel clamping. (2) Nephrotoxic folic acid-induced AKI: mice (female, 12–14 weeks old) received a single i.p. injection of folic acid at 250 mg/kg in 0.3 mol/L sodium bicarbonate or the vehicle. For therapeutic experiments, RR-11a was freshly dissolved in saline. Mice were administered an i.p. injection of 20 mg/kg RR-11a or the vehicle before ischemia. Mice were euthanized at the indicated times to collect serum samples and kidney tissues.

### Assessment of kidney functions and histology

Concentrations of BUN and serum creatinine were measured using a urea and creatinine assay kit (Nanjing Jiancheng Bioengineering Institute, Nanjing, China). Periodic acid-Schiff (PAS) staining (Solarbio, Beijing, China) was performed according to the manufacturer’s instructions. Immunohistochemistry and immunofluorescence staining were performed as described previously^27^. For immunohistochemistry, an antibody against CD3 (1:200, sc-20047; Santa Cruz Biotechnology, Inc., Dallas, TX) was used. Sections were counterstained with hematoxylin. Quantification was conducted in 15 randomly chosen fields (×400), and the percentages of the positively stained areas were calculated. For immunofluorescence staining, a primary anti-F4/80 antibody (1:200, ab6640; Abcam, Inc., Cambridge, MA) was used. Nuclei were stained with DAPI and the specimens were mounted in fluorescence mounting medium (Dako, Carpinteria, CA). The specimens were analyzed by confocal microscopy (FV1000, Olympus Corp., Tokyo, Japan). The number of F4/80^+^ macrophages was counted in 15 randomly chosen fields (×400).

### Evaluation of renal damage

PAS-stained kidney specimens were used to evaluate renal damage, tubular detachment, tubular dilation, protein cast, and the brush border damage were quantitated in 10 randomly selected fields (original magnification, ×200). Percentage of areas per whole section are presented. Evidence of tubular detachment, proximal tubular dilation, and brush border damage were scored from zero to 10 (0–1, none; 1–2, <11%; 2–4, 11–25%; 4–6, 26–45%; 6–8, 46–75%; 8–10, >75%)^40^. Results for each item were added to yield the ATN score.

### RNA extraction and quantitative real-time PCR

Total RNA was extracted using TRIzol Reagent (Invitrogen, Carlsbad, CA), according to the manufacturer’s instructions, and 2 μg RNA was reverse transcribed with TransScript First-Strand cDNA synthesis SuperMix (TransGen Biotech, Beijing, China). Quantitative PCR was performed with TransStart Top Green qPCR SuperMix (TransGen Biotech) in a CFXTM Real-Time Thermal cycler (Bio-Rad, Hercules, CA). Primers are described in the supplemental material. Data were analyzed by the 2^–ΔΔCt^ method, normalized to β-actin, and compared with controls.

### Mouse primary tubule isolation and cell culture

Kidneys were collected immediately after the mice were euthanized, and cortexes were minced and digested for 1 hour using 1 mg/ml collagenase IV (Sigma-Aldrich). The tissue homogenate was filtered through 40-μm and then 70-μm strainers (BD Falcon, San Jose, CA) twice. Samples were centrifuged to obtain a pellet containing renal tubules. Tubules were cultured in DMEM/F12 medium containing 25 ng/ml mouse epithelial growth factor (Sino Biological, Beijing, China). Human proximal tubular cell line HK-2 was purchased from the ATCC (Manassas, VA) and cultured in DMEM/F12 medium containing 10% FBS and 1% penicillin and streptomycin (Gibco, Gaithersburg, MD). Cells were incubated in a hypoxic (0.001% O_2_) chamber for 6, 12, and 24 hours to mimic ischemic conditions in vivo.

### Western blotting

Kidney tissues and cell pellets were homogenized in 1×radioimmunoprecipitation assay buffer containing protease inhibitor cocktails, and the insoluble residue was removed by centrifugation for 10 minutes at 12,000 × *g*. The protein concentration was determined using the Bradford assay (Thermo Fisher Scientific, Waltham, MA). Equal amounts of proteins were separated by 10–15% SDS-PAGE. Samples were transferred to polyvinylidene difluoride membranes (EMD Millipore, Billerica, MA), blocked with 5% dry skim milk, and incubated with primary antibodies including anti-legumain (1:1000; AF2058; R&D Systems, Minneapolis, MN), anti-legumain (1:5000, ab183028), anti-GPX4 (1:2000, ab125066), anti-IL-33 (1:1000, alx-804-840-C100, Enzo Life Sciences, Plymouth Meeting, PA), TNF-α (1:1000, sc-12744, Santa Cruz Biotechnology, Inc.), anti-CCL-2 (1:1000, sc-52701, Santa Cruz Biotechnology, Inc.), anti-phospho-MLKL (1:1000; ab196436; Abcam, Inc.), mouse anti-β-actin (1:5000, sc-47778, Santa Cruz Biotechnology, Inc.), rabbit anti-β-actin (1:1000, sc-1616-R), anti-LAMP1 (1:1000; sc-17768; Santa Cruz Biotechnology, Inc.), anti-HSP90 (1:1000; WL01763; Wanleibio, Shenyang, China), anti-HSC70 (1:1000, 10654-1-AP, ProteinTech Group, Chicago, IL, USA), anti-lamp-2a (1:1000, 66301-1-Ig, ProteinTech Group) or anti-caspase 3 (1:1000; 9662 s; Cell Signaling Technology, Beverly, MA) at 4 °C overnight. After washing with TBS/Tween, the membranes were incubated with a horseradish peroxidase-conjugated secondary antibody (Santa Cruz Biotechnology, Inc.). Blots were developed with an ECL kit (EMD Millipore).

### Detection of intracellular ROS

Two kinds of fluorescent probes were used to detect intracellular ROS in kidney tissues and cells, namely 5-(and-6)-carboxy-2′,7′-dichlorodihydrofluorescein diacetate (carboxy-H_2_DCFDA; Sigma-Aldrich), and dihydroethidium (DHE; Sigma-Aldrich). For DHE staining, cryosections were incubated with 10 μM DHE at 37 °C for 30 min and then observed by confocal microscopy to determine the percentage of the DHE-stained area. For carboxy-H2DCFDA staining, kidney tissues were homogenized with PBS and centrifuged. Then, the supernatant was collected and incubated with 10 μM H_2_DCFDA at 37 °C for 30 min. For ROS detection in cultured cells, the cells were seeded in a 96-well plate and incubated with H_2_DCFDA. Fluorescence was detected by a SpectraMax Microplate Reader (Molecular Devices, San Jose, CA) at 488 nm excitation and 525 nm emission.

### MDA assay

The relative MDA concentration in kidney tissues and cells was assessed with a lipid peroxidation MDA assay kit (Beyotime, Shanghai, China), according to the manufacturer’s instructions. Briefly, kidney tissues or cells were homogenized in lysis buffer, incubated with thiobarbituric acid at 100 °C for 15 min, centrifuged, and then absorbance was determined at 532 nm.

### Cell survival and LDH release assays

Cell survival was determined using a cell counting kit-8 (CCK-8) assay (Dojindo Laboratories, Kumamoto, Japan). mRTECs and HK-2 cells were seeded in 96-well plates at 3000 cells per well and treated with erastin or incubated under hypoxic conditions. Subsequently, 110 μl of fresh medium containing 10 μl CCK-8 solution was added to the cells, followed by incubation for 2 h (37 °C, 5% CO_2_). Absorbance at 450 nm was then measured. LDH release was detected using a lactate dehydrogenase (LDH) kit (Nanjing Jiancheng Bioengineering Institute). mRTECs were seeded in 6-well plates at 1 × 10^5^ cells per well and treated with erastin or incubated under hypoxic conditions. The culture supernatant was collected and the cells were treated with 1.5% Triton X-100, and then the cells and supernatant were incubated with coenzyme I and 2,4-dinitrophenylhydrazine for 15 min at 37 °C. Absorbance was then determined at 490 nm.

### Lysosome isolation

Lysosomes were harvested by homogenization and sequential centrifugation with a lysosome isolation kit (BestBio, Shanghai, China), according to the manufacturer’s protocol.

### Plasmid construction and transfection

Mouse legumain cDNA was amplified by reverse transcription-PCR and then ligated into pLV-EF1α-MCS-IRES-Bsd (Biosettia, San Diego, CA). mRTECs were transfected with the pLV-EF1α-legumain-IRES-Bsd plasmid. Cells were seeded in a 6-well plate containing medium without penicillin-streptomycin and then transfected using Lipofectamine™ 2000 transfection reagent (Invitrogen).

### Immunoprecipitation

Cell pellets were lysed in lysis buffer and incubated with an antibody against legumain (312114; Invitrogen) at 4°C overnight. Immunocomplexes were captured using protein G-sepharose (GE Healthcare, Buckinghamshire, UK) for 3–5 h at 4 °C. The beads were washed with PBS five times, and then a loading buffer was used to elute the immunocomplexes.

### Statistical analyses

Data were analyzed by GraphPad Prism, ver.7.0 (GraphPad Software Inc. La Jolla, CA) and shown as the mean ± SD. The unpaired Student’s t-test was used to compare two groups. ANOVA was used to compare more than two groups.

## Supplementary information

Supplemental figure 1

Supplemental figure 2

Supplemental figure 3

Supplemental figure 4

Supplemental figure 5

Supplemental figure 6

Supplementary Figure Legends
