# Supplementary figures and images for: Legumain promotes tubular ferroptosis by facilitating chaperone-mediated autophagy of GPX4 in AKI
Source: Cell Death Dis. 2021 Jan 11;12(1):65. doi: 10.1038/s41419-020-03362-4 (PMC7801434; doi:10.1038/s41419-020-03362-4)

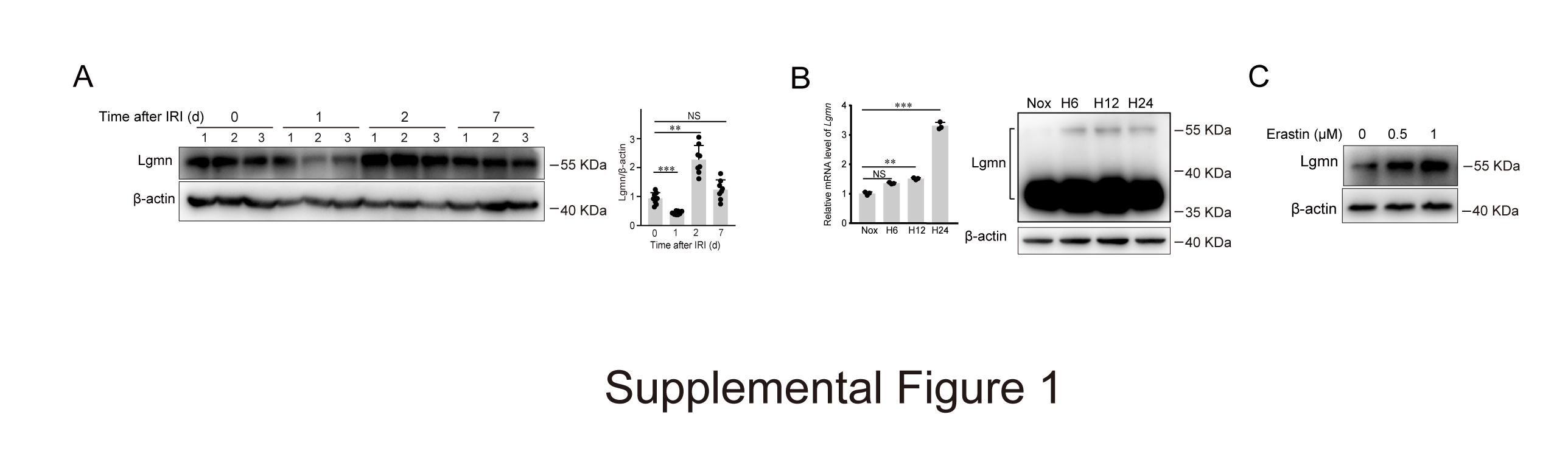

Supplement: Supplementary file 1 — Supplemental figure 1 [file 41419_2020_3362_MOESM1_ESM.tif]

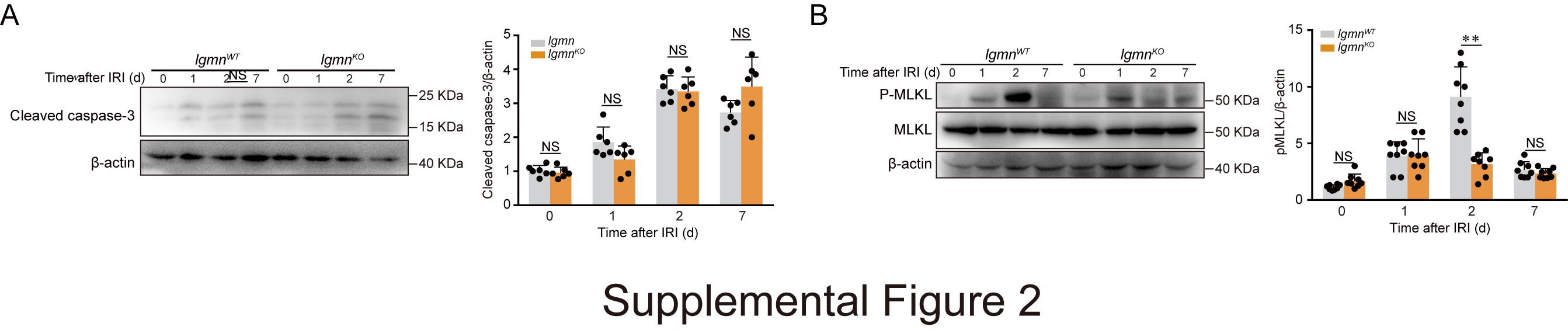

Supplement: Supplementary file 2 — Supplemental figure 2 [file 41419_2020_3362_MOESM2_ESM.tif]

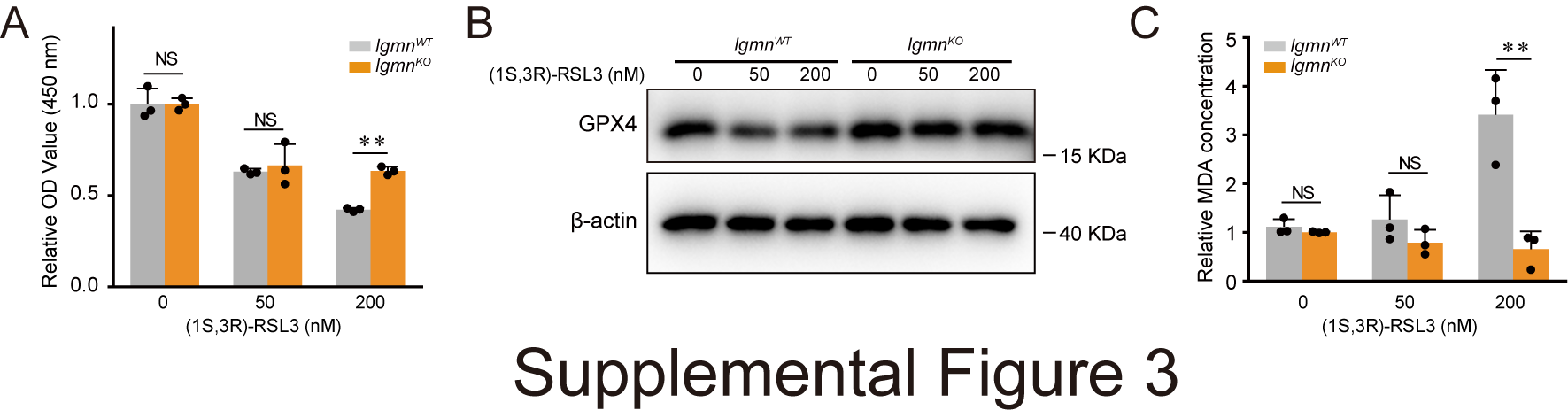

Supplement: Supplementary file 3 — Supplemental figure 3 [file 41419_2020_3362_MOESM3_ESM.tif]

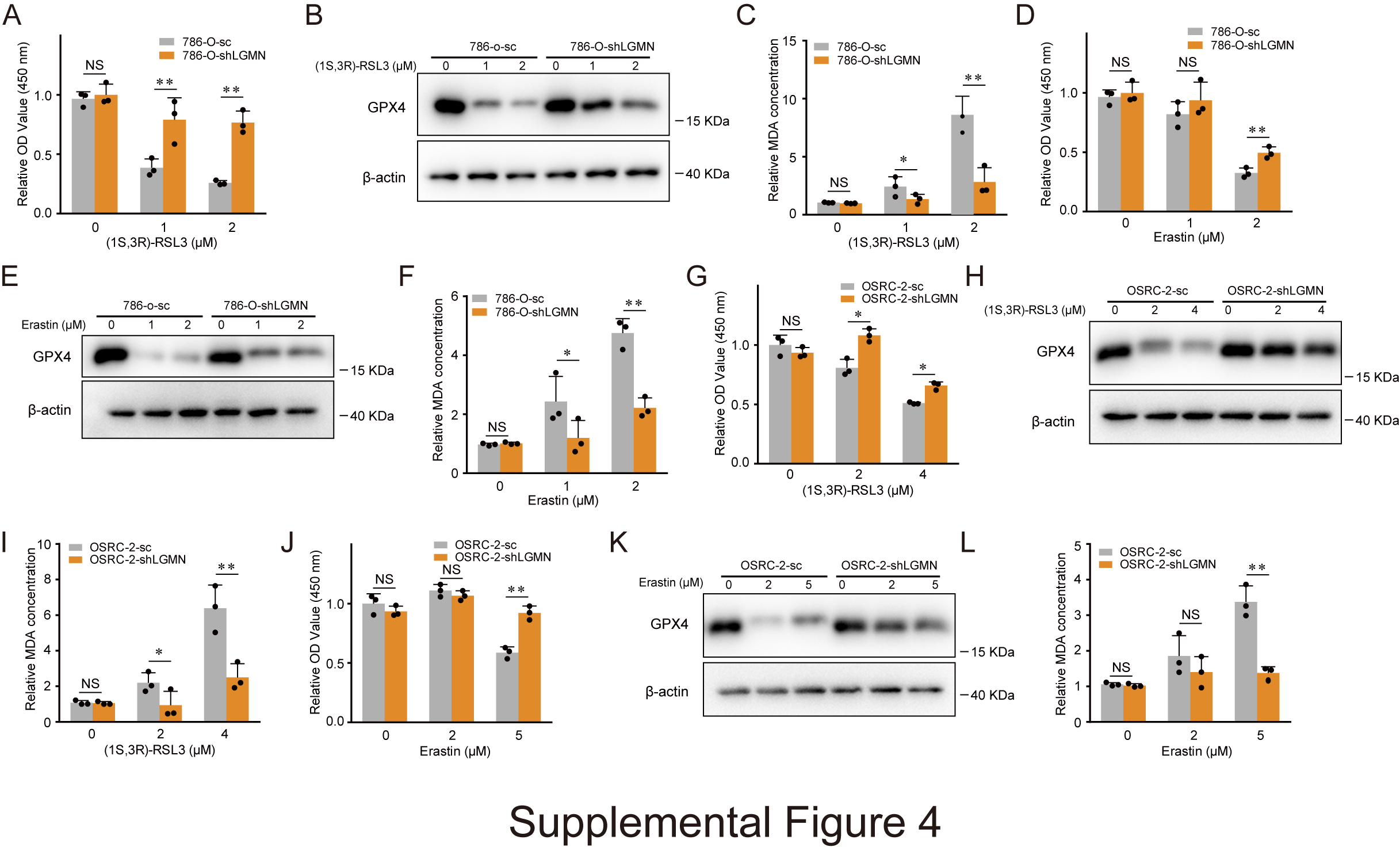

Supplement: Supplementary file 4 — Supplemental figure 4 [file 41419_2020_3362_MOESM4_ESM.tif]

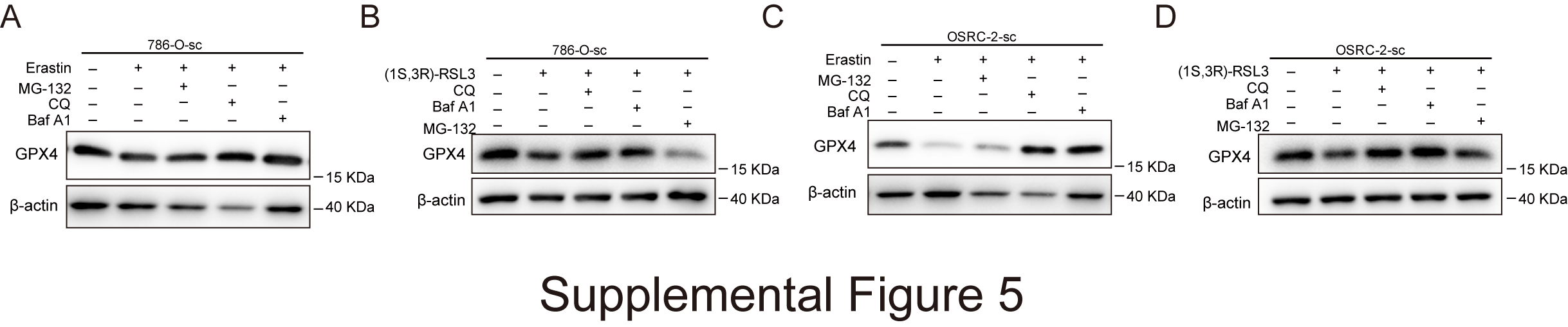

Supplement: Supplementary file 5 — Supplemental figure 5 [file 41419_2020_3362_MOESM5_ESM.tif]

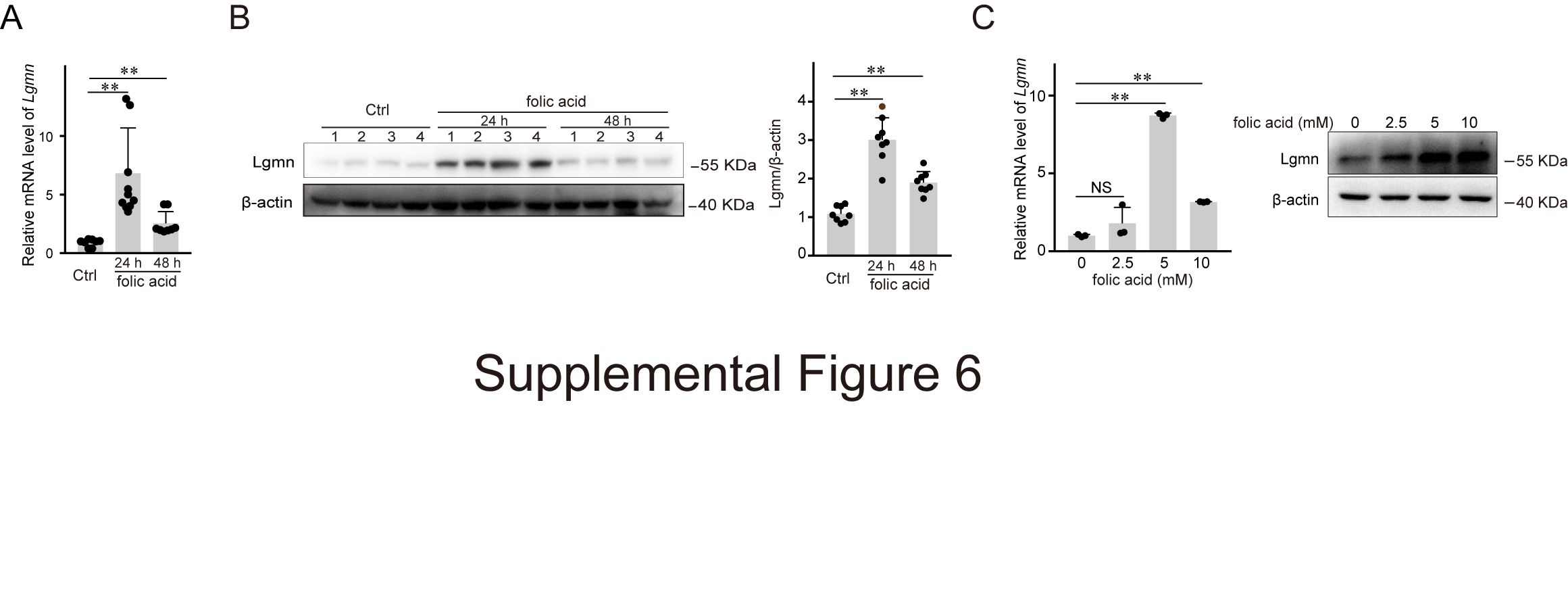

Supplement: Supplementary file 6 — Supplemental figure 6 [file 41419_2020_3362_MOESM6_ESM.tif]
