# Supplementary material for: Legumain promotes tubular ferroptosis by facilitating chaperone-mediated autophagy of GPX4 in AKI
Source: Cell Death Dis. 2021 Jan 11;12(1):65. doi: 10.1038/s41419-020-03362-4 (PMC7801434; doi:10.1038/s41419-020-03362-4)
Supplement: Supplementary file 7 — Supplementary Figure Legends [file 41419_2020_3362_MOESM7_ESM.docx]

**SUPPLEMENTAL MATERIALS**

**Extended Materials and Methods**

**Supplemental figure legends**

**Extended** **Materials and Methods**

**Primer sequences**

The following primers were used: mus-Legumain-forward (F): 5’-agaggatgtgactccagagaa-3’ and reverse (R): 5’-ccgtggtcggtgaagtaaat-3’, mus-β-actin-F: 5’- cagaaggagattactgctctggct-3’ and R: 5’-tactcctgcttgctgatccacatc-3’, mus-Acsl4-F: 5’- acttacctttggctcatg-3’ and R: 5’-cagtacagtacaatcaccct-3’, mus-Cox-2-F:5’- cacactctatcactggcacc-3’ and R: 5’-tccaggaggatggagttgtt-3’, mus-TNF-α-F: 5’-aaggccggggtgtcctggag-3’ and R: 5’-aggccaggtggggacagctc-3’, mus-CCL-2-F: 5’-cctgctgttcacagttgcc-3’ and R: 5’-tggacccattccttcttgg-3’, mus-IL-33-F: 5’-tgcaggaaagtacagcattca-3’ and R: 5’-ctcatgcagtagacatggca-3’, mus-IL-1α-F: 5’-tctgccattgaccatctc-3’ and R: 5’-atcttcccgttgcttgac-3’, mus-Kim-1-F: 5’-atcccatactcctacagact-3’ and R: 5’-ccaacatagaagccctta-3’, mus-NGAL-F: 5’-aaggcagctttacgatgt-3’ and R: 5’-tggttgtagtccgtggtg-3’, mus-Gpx4-F: 5’-aggcaggagccaggaag-3’ and R: 5’-ccttgggctggactttc-3’. Data were analyzed with a method of 2^–ΔΔCt^ normalized to β-actin and compared with controls.

**Supplemental figure legends**

**Supplemental Figure 1. Legumain expression in the IRI-induced AKI models and hypoxia- or erastin-stimulated mRTECs.** Kidney samples were from *lgmn*^WT^ mice before or after different time periods of IRI (n = 8). (**A**) Protein level of legumain in kidney samples assessed via western blot and quantification was shown in the right panel. mRTECs were isolated from *lgmn*^WT^ mice. (b) Isolated mRTECs were incubated under under normoxic or hypoxic condition for 6, 12, 24 hours. Quantification of *Lgmn* mRNA level by qPCR（left）and protein level of legumain was assessed via western blot (right). mRTECs were treated with different dose of erastin. (**C**) Protein level of legumain was assessed via western blot. All data are expressed as mean ± SD. ^**^ *P* < 0.01, ^***^ *P* < 0.001; student's t test.

**Supplemental Figure 2. Apoptosis and necroptosis in the IRI-induced AKI model.** Animal model and sample collection were the same as described in Fig 1. (**A**) Western blot using the antibody against caspase-3, Quantification of cleaved-caspase 3 presence in the right panel. (**B**) Western blot using the antibody against MLKL and p-MLKL. Quantification of p-MLKL presence in the right panel. All data are expressed as mean ± SD. ^**^ *P* < 0.01; two-way ANOVA.

**Supplemental Figure 3. Deletion of legumain inhibits (1S, 3R)-RSL3-induced ferroptosis in primary renal tubular Cells.** mRTECs isolated from *lgmn*^WT^ and *lgmn*^KO^ mice were treated with (1S, 3R)-RSL3 (50 nM, 200 nM) for 24 hours. (**A**) Cell survival was measured by CCK-8 assays. (**B**) Western blot assay of GPX4. (**C**) Lipid peroxidation levels were measured by MDA assays. Data were collected from three independent experiments. All data are expressed as the mean ± SD. ^**^*P* < 0.01; two-way ANOVA.

**Supplemental Figure 4. Downregulation of legumain inhibits ferroptosis in renal carcinoma cells.** Stable legumain knockdown and scramble control cells were established in 786-O and OSRC-2 cells. (**A-F**) 786-O scramble control (786-O-sc) cells and legumain knockdown (786-O-shLGMN) cells were treated with erastin or (1S, 3R)-RSL3 for 24 hours. (**A, D**) Cell viability was measured by CCK-8 assays. (**B, E**) Western blot assay of GPX4. (**C, F**) Lipid peroxidation levels were measured by MDA assays. (**G-L**) OSRC-2 scramble control (OSRC-2-sc) cells and legumain knockdown (OSRC-2-shLGMN) cells were treated with erastin or (1S, 3R)-RSL3 for 24 hours. (**G, J**) Cell viability was measured by CCK-8 assays. (**H, K**) Western blot assay of GPX4. (**I, L**) Lipid peroxidation levels were measured by MDA assays. All data are expressed as the mean ± SD. ^*^*P* < 0.05, ^**^*P* < 0.01; two-way ANOVA.

**Supplemental Figure 5 Legumain participates in lysosomal degradation of GPX4 during ferroptosis in renal carcinoma cells.** 786-O cells were pretreated with MG132 (100 nM), CQ (25 μM), and Baf A1 (50 nM) for 2 hours before treatment with 1 μM erastin or 1 μM (1S, 3R)-RSL3 for 24 hours. (**A-B**) Western blot using an antibody against GPX4. OSRC-2 cells were pretreated with MG132 (100 nM), CQ (25 μM), and Baf A1 (50 nM) for 2 hours before treatment with 2 μM erastin or 2 μM (1S, 3R)-RSL3 for 24 hours. (**C-D**) Western blot using an antibody against GPX4.

**Supplemental Figure 6. Legumain expression in folic acid-induced AKI model.** Kidney samples were collected from *lgmn*^WT^ mice after i.p. injection with folic acid for 24 and 48 hours (n = 8). (**A**) Quantification of *lgmn* mRNA expression by qPCR. (**B**) Protein level of legumain assessed via western blot. Quantification was shown in the right panel. mRTECs were isolated from *lgmn*^WT^ mice and treated with different dose of folic acid for 24 hours. (**C**) Quantified *lgmn* mRNA levels by qPCR (left) and protein level of legumain assessed via western blot (right). All data are expressed as mean ± SD. ^**^ *P* < 0.01; student's *t* test.
